# HCV Reactivation Risk in Patients Undergoing Treatment for Inflammatory Arthritis and Connective Tissue Diseases

**DOI:** 10.31138/mjr.030425.aic

**Published:** 2025-12-31

**Authors:** Maria Pappa, Theodoros Androutsakos, Anastasios Karamanakos, Evangelia Mole, Souzana Gazi, Fabiola Atzeni, Marco Sebastiani, Konstantinos D. Vassilakis, Charalampos Papagoras, Nikolaos Kougkas, George E. Fragoulis

**Affiliations:** 1 First Department of Propaedeutic and Internal Medicine, National and Kapodistrian University of Athens, Athens, Greece;; 2 Department of Pathophysiology, National and Kapodistrian University of Athens, Athens, Greece;; 3 Department of Rheumatology, “Evangelismos” Hospital, Athens, Greece;; 4 Department of Rheumatology, “KAT” Hospital, Athens, Greece;; 5 Rheumatology Unit, Department of Internal and Experimental Medicine, University of Messina, Messina, Italy;; 6 Rheumatology Unit, AUSL Piacenza, Italy;; 7 Department of Medicine and Surgery, University of Parma, Parma, Italy;; 8 First Department of Internal Medicine, University Hospital of Alexandroupolis, Democritus University of Thrace, Alexandroupolis, Greece;; 9 Department of Rheumatology, 4th Department of Internal Medicine, Aristotle University, Hippokration Hospital, Thessaloniki, Greece

**Keywords:** DMARDs, Hepatitis C, immunosuppressives, inflammatory arthritis, reactivation risk, rheumatic diseases

## Dear Editor,

Patients with Autoimmune Inflammatory Rheumatic Diseases (AIRD), who receive immunosuppressive or immunomodulatory treatments, have a significantly higher risk for opportunistic infections or exacerbation of chronic ones, including infectious hepatitis. In this regard, the management of AIRD patients with concomitant hepatitis C virus infection (HCV) is often challenging.^1^ Importantly, evidence on the safety of immunosuppressive/immunomodulatory treatments are mainly derived from patients with chronic HBV infection,^2,3^ whereas data on patients with co-existing HCV infection are limited, especially for newer targeted synthetic (ts-) or biologic (b-) disease modifying antirheumatic drugs (DMARDs).

HCV, unlike HBV, does not integrate into the host genome and therefore does not undergo true reactivation after successful treatment. Our aim is to describe the safety of immunosuppressive/immunomodulatory treatments in patients with AIRDs and active HCV infection and examine the frequency of HCV reactivation. In this case series, we included patients with AIRDs and confirmed HCV infection (HCV-RNA positive) followed in seven rheumatology centres in Greece and Italy. A total of 9 patients, 55% female, with a median (IQR) 63 (55–81) years of age and median (IQR) disease duration of 6.5 (4–13.5) years were identified and followed for 12 months after the documentation of HCV infection. Seven patients were diagnosed with inflammatory arthritis (IA), i.e. three patients with rheumatoid arthritis (RA), three with psoriatic arthritis (PsA) and one patient with axial spondylarthritis (AxSpA), while the remaining 2 were diagnosed with primary Sjögren’s syndrome and cryoglobulinaemic vasculitis, respectively. HCV RNA was measured at two timepoints: at the initiation of DMARD treatment and at 12 months follow-up. All patients received HCV treatment, including direct acting anti-virals (66.7%) and interferon alpha (IFN-a) (22.2%). The median (IQR) treatment duration was 3 (3–7) months; four patients had completed HCV treatment before starting any DMARD therapy, while the remaining five patients began HCV treatment and DMARD therapy simultaneously. Regarding anti-rheumatic treatments used during the one year of follow-up, hydroxychloroquine was prescribed in 22.2% of patients, 3 individuals (33%) were on immunosuppressants (including methotrexate and cyclosporine), while 55.5% used b/ts-DMARDs, including anti-TNF-a and anti-IL-17 or anti-IL-23 equally. The median (IQR) prednisolone dose was 5 (0–15) mg. Notably, despite the theoretical risk no cases of HCV reactivation were observed, as defined by increase in HCV RNA of at least one logarithm or increase in liver function tests (of at least two times) at 12 months follow-up, indicating no virological reactivation. No differences were observed between different DMARD regimens and treatment outcomes. The detailed characteristics are presented in **Table 1**.

**Table 1.** Descriptive characteristics of HCV- RNA positive patients.

	n=9
Age, years (median, IQR)	63 (55– 81)
Female, n (%)	5 (55)
Inflammatory Arthritis, n (%)	7 (77)
Disease duration, years (median, IQR)	6.5 (4–13.5)
HCV treatment, n (%)	9 (100)
*-Sofosbuvir/ledipasvir, n (%)*	3 (33.3)
*-Sofosbuvir/velpatasvir, n (%)*	2 (22.2)
*-IFN, n (%)*	2 (22.2)
*-Dasabuvir, n (%)*	1 (11.1)
*-other, n (%)*	1 (11.1)
HCV treatment duration, months (median, IQR)	3 (3–7)
Hydroxychloroquine, n (%)	2 (22.2)
(b-ts-)-DMARDs, n (%)	5 (55.5)
*-anti-TNF-a, n (%)*	2 (22.2)
*-anti-IL-17 or- Il23, n (%)*	2 (22.2)
*-CTLA4 inh, n (%)*	1 (11.1)
Immunosuppressants^ , n (%)	3 (33.3)
Current prednisolone dose (median, IQR)	5 (0–15)
HCV reactivation * , n (%)	0 (0)

^ Methotrexate, cyclosporine A

* HCV reactivation defined as an increase in HCV-RNA of at least 1 logarithm or an increase in liver function tests of at least two times the upper limit of normal

Current literature regarding the safety of tumour necrosis factor-alpha (TNF-α) inhibitors in HCV-infected rheumatic patients indicate an overall favourable safety profile, and report low reactivation rates under close monitoring.^4^ A multicentre study including psoriatic arthritis (PsA) patients with chronic HCV infection demonstrated that TNF-α blockers, such as etanercept and adalimumab, effectively reduced disease activity without evidence of HCV reactivation or significant liver function deterioration.^5,6^ A systematic review involving 153 patients found no significant hepatotoxicity or increase in HCV viral load in most cases.^7^ Etanercept, in particular, was associated with stable transaminase levels and viral load during treatment.^6,7^ Infliximab and adalimumab were also generally safe, although isolated cases of transient transaminase elevation were reported.^7^ Additionally, retrospective and real-world data confirm the argument that careful screening and monitoring for HCV reactivation can mitigate risks associated with anti-TNF therapies.^8^ Regarding csDMARDs, data are very limited, with only one study reporting HCV reactivation in RA patients receiving these drugs. In this setting HCV reactivation appears to be less than 4%.^8^ Nonetheless, it is essential to recognize that patients with detectable HCV-RNA carry a plausible risk of viral reactivation, especially in the context of potent immunosuppression. Herein, we corroborate the findings from previous studies regarding csDMARDs and anti-TNF therapies and we report safety data for other bDMARDs, especially IL-17 and IL-23 inhibitors.

In conclusion, anti-rheumatic drugs seem to be safe for patients with AIRDs and concomitant HCV infection when used under vigilant clinical supervision. The risk of HCV reactivation is low, but appropriate baseline evaluation and ongoing monitoring are essential to ensure favourable outcomes.

## Data Availability

Data are available upon reasonable request.
